# Case Report: Resolution of high grade anal squamous intraepithelial lesion with antibiotics proposes a new role for syphilitic infection in potentiation of HPV-associated ASCC

**DOI:** 10.3389/fonc.2023.1226202

**Published:** 2023-10-03

**Authors:** A. Ranabhotu, N. Habibian, B. Patel, E. Farrell, J. Do, S. Sedghi, L. Sedghi

**Affiliations:** ^1^ Gatroenterology Associates of Central Georgia, Macon, GA, United States; ^2^ Mercer University School of Medicine, Macon, GA, United States; ^3^ Advanced Pathology Solutions, Department of Gastroenterology, Little Rock, AR, United States; ^4^ Department of Oral and Craniofacial Sciences, University of California San Francisco School of Dentistry, San Francisco, CA, United States

**Keywords:** coinfection, syphilis, human papillomavirus (HPV), anogenital squamous cell carcinoma (ASCC), anal intraepithelial lesions

## Abstract

**Introduction:**

Human Papillomavirus (HPV) is the primary risk factor for the development of anal intraepithelial neoplasia (AIN) and is a leading risk factor for anogenital squamous cell carcinoma (ASCC). Despite common shared risk factors for both HPV and syphilis, co-infection is not well documented, and the role of syphilitic infection in HPV-associated AIN and ASCC potentiation is not defined.

**Case description/methods:**

A 72-year-old single male presented with complaints of mild rectal pain and intermittent rectal bleeding. A flexible sigmoidoscopy was performed, and a firm 4.5cm x 3cm perianal mass was detected and superficially biopsied. Pathology findings demonstrated evidence of a high grade squamous intraepithelial lesion (HGSIL, AIN II/III/AIS) with viral cytopathic effect, consistent with HPV infection. Much of the biopsied lesion showed acanthotic squamous mucosa with intraepithelial neutrophils and abundant submucosal plasma cells, suggesting possible syphilitic involvement. Subsequent immunohistochemical staining for p16 as a surrogate marker for HPV was positive, as was an immunohistochemical stain for spirochetes, supportive of co-infection with *Treponema pallidum pallidum* (*T. pallidum)*, the causative agent in venereal syphilis. The patient was referred to an infectious disease specialist for syphilitic infection and was treated with penicillin with surprisingly complete resolution of the lesion. EUAs were performed 2- and 3-months following treatment without lesion recurrence. However, one year following diagnosis, a flexible sigmoidoscopy revealed a 5 mm recurrent HPV-related low-grade AIN 1 lesion at the dentate line.

**Discussion:**

Resolution of the lesion by antibiotic treatment for syphilitic infection suggested that co-infection by *T. pallidum* may potentiate HPV-associated squamous cell carcinoma based on histological findings. Findings from this case, as well as a review of bacterial involvement and potentiation in various cancers, are reviewed here. Such findings offer new insight regarding the role of STI-associated bacteria and HPV co-infection in the establishment of AIN and may additionally propose new treatment modalities for ASCC.

## Introduction

Anal squamous cell carcinoma (ASCC) is the most common cancer type of the anal region. ASCC cancer originates in the squamous cells, which make up both the lining of the anal canal and the anal margins (1). Anal cancer is a common disease that is a public health concern worldwide, with an increased incidence by two- to four-fold in Europe, Australia, and the United States among both men and women within the past three decades (2, 3). Anal cancer accounts for less than 1% of all new cancer diagnoses and for about 4% of all gastrointestinal tract cancers (4, 5). In 2020, anal cancer accounted for approximately 50,685 new cases and 19,293 deaths worldwide (6). In the United States alone, the American Cancer Society recorded 8,580 new adult cases (2,960 male and 5,620 female) and 1,160 deaths (480 male and 680 female) due to anal cancer in 2018 (7). In 2022, anal cancer was projected to account for 9,440 new adult cases (3,150 male and 6,290 female) and an estimated 1,670 deaths (740 male and 930 female) in the United States (6). These numbers represent a 10% increase in new cases and a 44% increase in anal cancer deaths from 2018 to 2022 (7). The 5-year survival rates for anal cancer has improved in the last couple of decades and demonstrates a favorable prognosis, that being ~75% (8). However, increased rates for human papillomavirus (HPV) associated anal cancer showcases the need for the discovery of novel therapeutic approaches (9).

HPV, one of the most common sexually transmitted infections (STI) in the United States, is the primary risk factor for the development of ASCC and its precursor lesion, anal intraepithelial neoplasia (AIN), especially with regard to the HPV16 and HPV18 subtypes (4, 10). Factors that increase the risk of HPV infection leading to malignancy include anal-receptive intercourse, high lifetime number of sexual partners, human immunodeficiency virus (HIV) infection, prior history of anogenital warts, lower genital tract malignancies, a history of other HPV-related cancers, autoimmune disorders, smoking, and history of transplantation/chronic immunosuppression (4, 5, 10–13). Results obtained from published studies from the 1990s showed that combined chemoradiotherapy was the backbone for the initial treatment (8, 14–16). Today, three-quarters of patients with ASCC receive chemoradiotherapy as their primary treatment option. Some of the morbidities associated with chemoradiotherapy include increased long-term cardiovascular toxicity (17), increased risk of infection, bruising and bleeding, nausea and vomiting, fatigue, and hair loss (18). Surgical treatment options are also available and remain the standard of care for recurrent and residual disease (19). Significant morbidity of anal stenosis, wound healing, and incontinence are common outcomes on the surgical route (20). GI adverse events, especially fecal incontinence, and sphincter insufficiency are some of the more common morbidities associated with anal cancer in general (21). Efficacy of treatment via chemoradiotherapy compared to surgical intervention has demonstrated that complete chemoradiotherapy has favorable long-term results (22). Early diagnosis and treatment are the best way to improve quality of life for patients with ASCC.

Treatment for anal intraepithelial lesions includes topical, ablative, and surgical approaches, where topical is generally the best option. All these treatment options are followed by active surveillance due to the high recurrence rate of AIN. Topicals, such as 5-fluorouracil, are applied either to the entire anal canal or the specific lesion and usually require 1-2 treatments. Topical treatment has demonstrated efficacy for complete resolution (or regression of high-grade lesions to low grade lesions) in 71%-79% of cases. Ablative therapy is effective only in patients with HIV (79% disease regression), while surgical excision of lesions is rarely used today due to its high morbidity and recurrence rate (23). Regular surveillance of patients with AIN, including digital rectal exam, anoscope, and application of Lugol’s solution, every 3 to 6 months is recommended by the American Society of Colon and Rectal Surgeons (24).

Despite common shared risk factors for both HPV and syphilis, co-infection by HPV and *T. pallidum*, the causative pathogen in syphilis, is not well documented. As such, the role of syphilitic infection in HPV-associated AIN and ASCC potentiation is not well-documented. However, co-infection by HPV and syphilis has been observed previously (25, 26). For example, a study conducted by the Sexually Transmitted Infection Ambulatory Clinic at the Santa Casa de Misericordia Hospital in Rio de Janeiro, Brazil demonstrated that 15.9% of patients were co-infected with HPV and syphilis (27). Here, we report a similar case of co-infection by HPV and *T. pallidum* as a fortuitous pathological finding in a biopsied AIN.

## Case description

**Figure 1:**
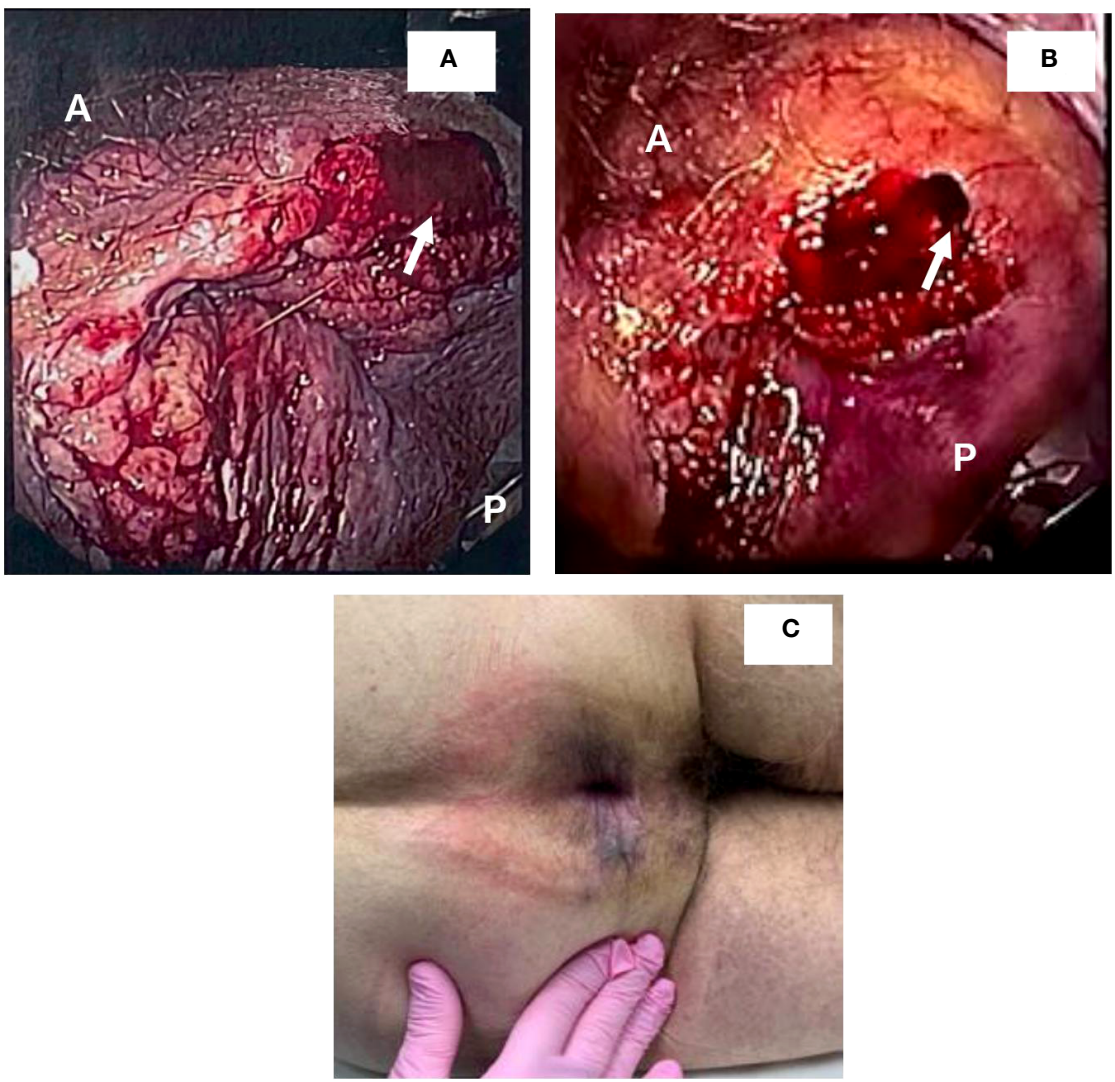
Detection and resolution of anal mass. **(A, B)** demonstrate views detected during sigmoidoscopy. A and P denote anterior and posterior positions, respectively. Arrows demonstrate areas that were biopsied. **(C)** demonstrates lesion resolution upon anal exam following treatment with penicillin.

A 72-year-old single male with a history of esophageal resection for Barrett’s esophagus with high-grade dysplasia and stage 1A esophageal cancer (without chemotherapy, with apparent cure, and no recurrence to date), presented with complaints of mild rectal pain and intermittent rectal bleeding. Three weeks later, a flexible sigmoidoscopy was performed, and a firm perianal mass was detected and superficially biopsied (**Figure 1A**). The specimen—multiple fragments of tan tissue that measured from less than 1mm to 2mm—was stored in a formalin-filled container prior to processing and was then submitted into a single cassette for processing. Technical portions of these services (grossing, processing, embedding, microtomy, and staining) were performed at Gastroenterology Associates of Central Georgia, LLC, CLIA #11D2015828. Professional portions of these services (all staining and immunohistochemistry) was performed at Advanced Pathology Solutions, Dept. of Gastroenterology CLIA # 04D2037153 in North Little Rock, Arkansas.

**Figure 2:**
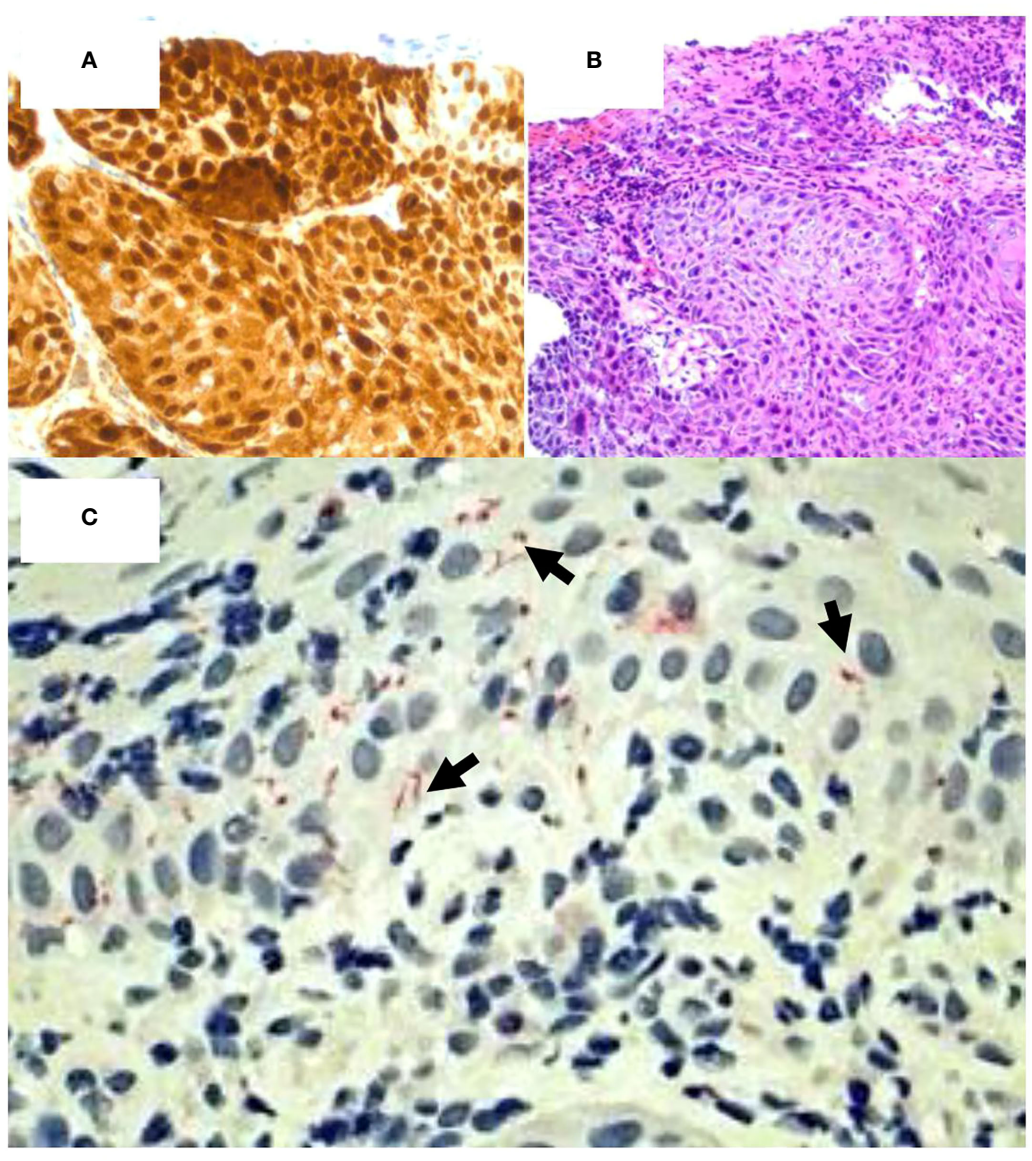
Coinfection of HPV and *T. pallidum* in high- grade squamous intraepithelial lesion. **(A)** p16 staining demonstrates HPV positivity. (Mag=20X). **(B)** H&E staining of p16 positive tissue fragment confirms HPV infection. (Mag=20X). **(C)** Spirochete stain (red) confirms the presence of syphilitic coinfection with HPV. (Mag=40X).

The mass measured 4.5cm x 3cm, and pathological findings revealed the lesion to be high grade squamous intraepithelial lesion (HGSIL, AIN II/II/AIS) with viral cytopathic effect, consistent with HPV infection (**Figures 2A, B**). HPV diagnosis was confirmed using a p16 immunohistochemical stain (**Figures 2A, B**); the efficacy of this technique has proven comparable to the historically used 16S rRNA sequencing (28–30). Due to the firmness of the lesion and the superficiality of the biopsy, it cannot be discounted that the patient may have had squamous cell carcinoma at deeper levels. Much of the biopsied lesion demonstrated acanthotic squamous mucosa with intraepithelial neutrophils and abundant submucosal plasma cells, consistent with pathological indicators of syphilitic involvement. An RPR/FTA-ABS serology test was performed; the results were 1:128 and showed positive reactivity, respectively. Additionally, immunohistochemical stain (rabbit polyclonal antibody, BioCare Medical) for spirochetes (**Figure 2C**) was positive. These findings—including presence of an anal lesion, histological confirmation of spirochete infection, and additional serological confirmation—is consistent with primary syphilitic infection. No systemic manifestations of secondary or latent syphilis were observed.

Of note, a PET and CT scan 1 year prior to diagnosis as well as a CT scan 7 months following diagnosis were negative for relevant findings to AIN. Furthermore, the patient denied any sexual history for the past 20 years; however, a colonoscopy performed one year prior to the diagnosis revealed a normal morphology, suggesting an acquired primary syphilis infection during the time between the colonoscopy and diagnostic sigmoidoscopy.

**Figure 3:**
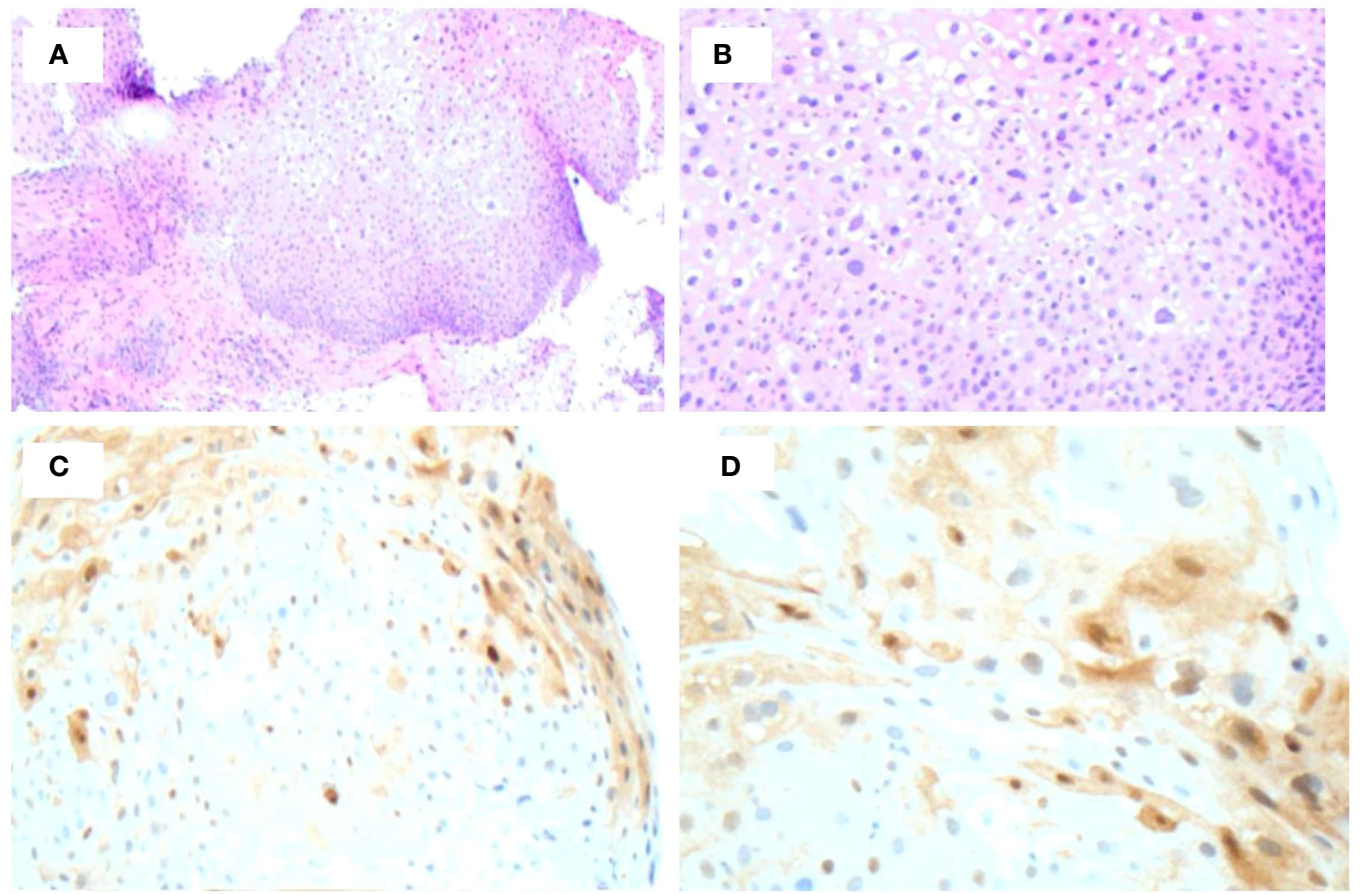
Biopsy of recurrent lesion one year following initial detection. **(A, B)** H&E staining of lesion demonstrates low-grade dysplasia. **(C, D)** p16 immunohistochemical stain demonstrates focal p16 positivity indicative or AIN 1.

Following the procedure, the patient was checked for CMV, HSV1/2, HIV, and hepatitis panels. immunohistochemical stains for CMV and HSV1/2 were negative. Labs demonstrated hepatitis A antibody negative, hepatitis B surface antigen negative, hepatitis B core antibody IgM negative, hepatitis C virus antibody negative, and HIV non-reactive. The patient was referred to an infectious disease specialist and syphilitic infection was treated with 3X doses of 2.4 MU Bicillin LA administered intramuscularly for three consecutive weeks at 7-day intervals. A total of 4 mL of Bicillin LA was injected with 2 mL on the left buttock and 2 mL on the right buttock each week. The RPR titer came down from 1:128 to 1:16 following treatment. Upon follow up with gastroenterology, remarkable and surprising resolution of the suspected HPV actuated dysplastic lesion was observed (**Figure 1B**). Two subsequent EUAs were performed, 2- and 3- months following treatment, and were evaluated as normal with complete resolution of the lesion (**Figure 1C**). However, a sigmoidoscopy performed one year later revealed a recurrent 5mm AIN 1 lesion at the dentate line which was promptly removed. The lesion was evaluated using Hematoxylin and Eosin stain (**Figures 3A, B**) as well as p16 immunohistochemical stain, which showed focal p16 positivity indicating low grade dysplasia/AIN 1 (**Figures 3C, D**). No treatment was required for the recurrent lesion. A follow-up flexible sigmoidoscopy was scheduled one year following lesion resolution; however, the appointment was cancelled. Currently, the patient is being contacted to reschedule the flexible sigmoidoscopy.

## Discussion

### Bacterial potentiation of cancer

The resolution of the lesion by antibiotic treatment for syphilitic infection suggests that co-infection by *T. pallidum* may potentiate HPV-associated squamous cell carcinoma, as based on histological findings. Findings of co-infection by HPV and *T. pallidum* with specific regard to ASCC are limited. As such, a review of bacterial involvement and potentiation in various cancer subtypes, along with associated treatment modalities, is presented here considering the findings from this case.

Bacterial infections may contribute to the development of cancer through the production of toxins, interrupting cellular signaling, altering DNA, and interfering with the normal functioning of the immune system resulting in inflammation (31, 32). Numerous bacterial strains are related to cancers, such as *Citrobacter rodentium* in colorectal cancer, *Helicobacter pylori* in gastric cancer, *Campylobacter jejuni* in small intestinal lymphomas, *Salmonella typhi* in hepatobiliary carcinomas, *Mycobacterium tuberculosis* in lung cancer, and *Chlamydia psittaci* in ocular lymphomas (33–38). The idea of bacterial involvement in the potentiation of cancer was largely neglected until 1890, when Dr. William Russell suggested the causal relationship between bacteria and cancer (39), and supporting discoveries were made throughout the twentieth century (40, 41). In fact, it has been shown that specific tumors have distinct bacterial compositions, and these specific compositional differences can be seen in benign versus metastatic disease of the same tissue (42, 43). The microbial DNA of these bacteria can be sampled, analyzed, and then used to diagnose whether an individual has cancer, and even what specific type (44). It has even been shown that there are different microbiome compositions in underlying versus overlying breast tissue (42).

Potentiation of oral squamous cell carcinoma (OSCC) by bacteria has also been documented. OSCC has been identified as a consequential outcome to infection by *Fusobacterium nucleatum* and *Porphyromonas gingivalis*, both putative pathogens in periodontal disease (45). This association was demonstrated via utilization of a mouse model, in which administration of *F. nucleatum* and *P. gingivalis* increased carcinogenesis (46). In addition to OSCC, *P. gingivalis* has been shown to play a role in various digestive tract tumors, and *F. nucleatum* is associated with colorectal carcinoma (47, 48). Colorectal carcinoma, also known as colorectal cancer (CRC), is influenced by bacteria residing within the oral cavity, or the oral microbiota (49). Most notably, disruption of the oral microbiota may induce dysbiosis among the intestinal microbiota, which may subsequently lead to CRC (49, 50). The oral microbiota in CRC is distinct, and therefore, profiling the oral microbiome could be an effective screening method for CRC (51). Cervical squamous cell carcinoma (CSCC) is another squamous cell carcinoma, and the risk of developing CSCC is increased by *Chlamydia trachomatis* infection, a bacterial infection (52). Although HPV is one of the top causes of CSCC, multiple studies have shown that *Chlamydia trachomatis* infection is a significant risk factor that can further potentiate the development of CSCC (52–54).

### Treatment of bacterial infection and improvement of cancer

Treatments for squamous cell carcinoma associated with HPV are typically unimodal or multimodal, depending on how early detection is made. HPV-associated head, neck, and oral squamous cell carcinomas are typically treated with surgery and aggressive radiotherapy or chemotherapy, and commonly accompanied by antibiotics for presumptive infection by pathogens associated with sexually transmitted infection (55–57). When antibiotics are administered, screening for sexually transmitted infection is not typically performed due to high efficacy. Antibiotics administration can range, but most administrations include quinolones or cotrimoxazole (58). Due to lack of screening, possible co-infection by STI-associated bacteria is typically undocumented. As a result, any synergistic benefits to STI-associated SCCs via treatment with antibiotics typically goes undocumented. With regard to CRC, the microbiome plays a pathologically unclear role in the recurrence and metastasis after surgical treatment (59) due to the wide variety of contributing bacteria (60, 61). Thus, specific bacterial identification is needed to mechanistically understand the improvement or worsening of squamous cell-associated cancers with presence of bacterial species.

*Limitations in STI reporting in elderly populations.* Cooperation in sexual reporting is often hindered among elderly populations due to (1) cultural and societal bias, (2) perceived stigma, embarrassment, and discrimination, (3) educational and training limitations among healthcare professionals, and the (4) quality of relationships shared among patients and healthcare professionals (62). In the described case, the patient denied having any sexual activity for 20 years. However, a colonoscopy performed one year prior to syphilitic and HIV-associated AIN demonstrated normal morphology. This observation possibly signifies that acquisition of syphilitic infection may have occurred during the one-year interval. HPV infection may have been acquired during this time also, or alternatively may have been latent and subsequently reactivated (63). There is increased evidence of acute STIs in the elderly (64); this may be due to multiple factors including loss of spouse (65), high risk sex (66), immunosuppression (67), and erectile dysfunction drugs (68). The CDC reports that STI incidence more than doubled in U.S. adults 65 years and older between 2007-2017. More specifically, rates of syphilitic infection increased nearly four-fold within this ten-year period, from 91 cases in 2007 to 349 cases in 2017 (69). This denial of recent sexual history may also be explained by susceptibility of self-reported sexual activity being under-reporting bias due to perception of social desirability. As such, self-identified sexual orientation may not serve as a reliable record of actual sexual preferences (63, 70).

## Conclusion

Co-infection of *T. pallidum* and HPV-associated cancers may lead to synergistic effects that potentiate symptoms and/or impact treatment in ASCC. Here, the identification of *T. pallidum* allowed for targeted antibiotic intervention by penicillin that correlated with the improvement of the high grade squamous intra-epithelial lesion (HGSIL). Literature review indicates these synergistic interactions may be more common than initially thought but are typically left uncharacterized and unstudied due to generalized antibiotic administration. This case study indicates a clinical need for STI testing and follow up care in patients afflicted by HPV-associated cancers and other metastases.

## Data availability statement

The raw data supporting the conclusions of this article will be made available by the authors, without undue reservation.

## Ethics statement

This research was conducted with the informed consent of all participants. The patients/participants provided their written informed consent to participate in this study. Written informed consent was obtained from the individual(s) for the publication of any potentially identifiable images or data included in this article. Written informed consent was obtained from the participant/patient(s) for the publication of this case report.

## Author contributions

SS treated the patient and collected the sample. SL worked on the case description and figures. SS, HN, PB, FE, DJ, and RA wrote the manuscript. All authors contributed to the article and approved the submitted version.
